# Oral Health and Sociodemographic Aspects in Transgender People: A Descriptive Exploratory Study

**DOI:** 10.1111/joor.13973

**Published:** 2025-04-17

**Authors:** Alex Moreira Mélo, Melissa de Oliveira Melchior, Fabiane Carneiro Lopes‐Olhê, Júlia Kefalás Troncon, Manoel Damião Sousa‐Neto, Laís Valencise Magri, Lúcia Alves da Silva Lara, Jardel Francisco Mazzi‐Chaves

**Affiliations:** ^1^ Department of Restorative Dentistry, School of Dentistry of Ribeirão Preto University of São Paulo São Paulo Brazil; ^2^ Department of Gynecology and Obstetrics, School of Medicine of Ribeirão Preto University of São Paulo São Paulo Brazil

**Keywords:** gender‐affirming hormone therapy, oral health, periodontal disease, sociodemographic factors, temporomandibular disorders, transgender

## Abstract

**Objectives:**

This study evaluated the oral health condition of transgender (trans) men and women before starting gender‐affirming hormone therapy (GAHT), focusing on the prevalence of periodontal disease and temporomandibular disorder (TMD) as well as the influence of alcohol consumption and anxiety.

**Materials and Methods:**

The study was a cross‐sectional, descriptive exploratory study conducted with transgender individuals over the age of 18 from the Gender Incongruence Clinic (AING) at Ribeirão Preto Medical School (FMRP‐USP). The study was approved by the Research Ethics Committee of the School of Dentistry of Ribeirão Preto, University of São Paulo (FORP/USP) (CAAE No. 02198018.5.0000.5419) and the Research Ethics Committee on Human Beings of the Ribeirão Preto Medical School (HCRP/FMRP) (CAAE No. 59992522.1.3001.5440). All volunteers provided informed consent before participation. Inclusion criteria consisted of individuals over 18 years of age, patients of the AING, who were hormone‐free or had not used hormones for at least 6 months (for trans women), and those with no prior hormone use (for trans men). Exclusion criteria included individuals with cognitive limitations that could hinder comprehension of the research instruments, those who had used analgesics, non‐steroidal anti‐inflammatory drugs (NSAIDs) or opioids within 24 h before the assessments, and individuals with reports of head and neck trauma, a history of plastic surgeries or orofacial harmonisation procedures or those undergoing orthodontic treatment or extensive oral rehabilitation. Each participant completed a sociodemographic questionnaire and was assessed for periodontal health using the Periodontal Screening Recording (PSR), TMD using DC/TMD criteria and anxiety levels with the GAD‐7 scale. Data analysis included descriptive statistics, chi‐squared, *T*‐test (*p* < 0.05) and multivariate logistic regression.

**Results:**

A total of 54 trans individuals over the age of 18 from the Gender Incongruence Clinic (AING) at Ribeirão Preto Medical School (FMRP‐USP) were invited, with 45 participants completing the study (24 trans men, 21 trans women). High levels of gingivitis and plaque accumulation were found in all participants, with no significant gender differences (*p* > 0.05). TMD was present in 83.33% of trans men and 47.62% of trans women, with a significantly higher incidence among trans men (*p* = 0.046). Alcohol consumption (*p* = 0.049) and anxiety (*p* = 0.043) were significantly associated with TMD, especially among trans men 1.624 [Confidence Interval (95% CI): 1.006–2.621].

**Conclusions:**

The study revealed alarming levels of gingivitis across all participants, highlighting the need for periodontal intervention. Over half of the participants exhibited TMD, with trans men showing a higher occurrence. TMD was significantly associated with alcohol consumption and anxiety.

**Clinical Relevance:**

The findings emphasise the need for integrated dental and mental health care in transgender populations, particularly for the prevention and management of TMD and periodontal disease.

## Graphical abstract

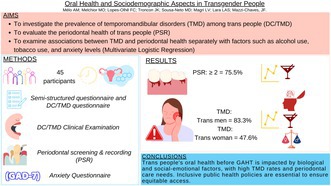

## 1 Introduction

This introduction outlines the key areas addressed in this study, beginning with the definition of a transgender person and the research on hormone treatment as a central component of gender‐affirming care. It then shifts focus to less explored but equally critical topics, including barriers to healthcare access, the effects of stress and minority stress, and the influence of psychosocial factors on oral health outcomes. Together, these factors frame the unique challenges faced by transgender individuals in achieving comprehensive health care. While hormone treatment has received considerable research attention, other important aspects, such as healthcare barriers, social stressors and their influence on health outcomes, remain underexplored. These factors profoundly impact the general and oral health of transgender individuals, emphasising the need for a broader perspective that goes beyond biological considerations. This manuscript seeks to fill these gaps by evaluating the oral health conditions of transgender individuals before initiating gender‐affirming hormone therapy, with a particular focus on the biopsychosocial influences shaping these outcomes.

A transgender (trans) person is someone whose gender identity does not align with the gender assigned to them at birth [1, 2]. Gender identity reflects how a person perceives and experiences their gender [3]. The ICD‐11 replaced “gender identity disorder” with “gender incongruence” to de‐pathologize trans identities, shifting the focus from “dysphoria” to “incongruence” [4]. Gender‐affirming hormone therapy (GAHT) allows trans individuals to adjust their body characteristics to align with their identified gender as desired. It constitutes is an important component of the gender‐affirming process [5]. However, this population often faces discrimination and stigmatisation, which can create significant barriers to accessing GAHT. These barriers include insufficient healthcare professional training, gaps in continuing education programs, financial limitations and the absence of a social support network [6].

Sex steroids used in GAHT can affect the stomatognathic system and oral health [7, 8], as evidenced by the influence of hormones on oral conditions in cisgender individuals [8]—those whose gender identity aligns with their biological sex [2]. For example, studies indicate that cisgender women experience higher rates of temporomandibular disorders (TMD) and periodontal diseases compared to cisgender men [8, 9, 10, 11], with hormonal factors such as oestrogen levels playing a role in these differences [10, 11, 12, 13]. Hormonal fluctuations, particularly those involving oestrogen, may increase the prevalence of TMD and contribute to periodontal inflammation [8, 10, 11]. Literature suggests that sex hormones influence oral tissue responses to bacterial stimuli, potentially exacerbating periodontal disease [14, 15]. Studies involving trans individuals on hormone therapy have highlighted the need for periodontal interventions, as increased levels of gingivitis and periodontitis have been observed [16, 17]. Additionally, exogenous oestrogen use has been linked to an increased risk of TMD [18], and polymorphisms in oestrogen receptors may heighten susceptibility to TMD in cisgender women [19]. Hormonal effects, especially related to oestrogen, may also affect pain sensitivity and modulation [20, 21], which could influence TMD development and other oral health concerns.

In addition to the influence of hormones, it is essential to highlight the healthcare access difficulties faced by trans people, who often encounter significant barriers when seeking health care, which reflects their social vulnerability [22, 23]. These challenges justify public inclusion ordinances and are a critical factor to consider. Self‐identification as trans is not necessarily linked to hormone use, and many may identify as trans without undergoing GAHT. Therefore, it is important to assess this population's oral health before initiating hormone therapy to better understand health conditions beyond hormonal impact. Studies show that socially vulnerable groups are more likely to develop periodontal diseases and exhibit signs and symptoms of TMD [24, 25]. Oral health should be approached from a biopsychosocial perspective, considering the connection between general and dental health.

Trans individuals also experience a higher prevalence of emotional disorders [26, 27], primarily due to the social stressors associated with their gender identity [28, 29]. This ‘minority stress’, stemming from discrimination and stigmatisation, can increase the risk of oral diseases influenced by stress and anxiety [30]. TMD, in particular, is directly associated with psychological factors such as anxiety and depression [30]. In addition to emotional stressors, trans people are more likely to engage in harmful substance use, such as smoking, alcohol consumption and drug use, at rates higher than their cisgender counterparts [31, 32]. These factors may contribute to increased oral health problems in this population, exacerbated by the lack of knowledge, stigmatisation and transphobia of healthcare professionals, often resulting in delayed treatment and diagnosis [16, 33]. Therefore, addressing the oral health of trans people must crucially encompass social and emotional dimensions, not just hormonal aspects.

There is a gap in epidemiological data on the oral health of the trans population [34], reflecting a lack of comprehensive dental care before and during the GAHT phase, as well as the absence of public policies for training qualified professionals [35, 36, 37]. Gender identity influences social stratification, exposing transgender individuals to risk factors. Understanding how social determinants of health (SDH) impact overall and oral health is crucial [38]. Besides hormonal factors, stress and anxiety play a role in the multifactorial aetiology of oral conditions. Therefore, oral health assessments should comprehensively consider the trans population, not merely those using hormones. A prior analysis of hormonal influence can clarify health conditions before hormone intervention. This study aims to evaluate the sociodemographic and oral health of trans women, trans men and non‐binary individuals before starting GAHT, hypothesising that even before GAHT, oral conditions may be exacerbated by frequent exposure to social and emotional stressors, such as discrimination and stigma, which can lead to delayed treatment and negatively affect both oral and general health.

## 2 Methodology

### 2.1 Ethics Statement

This descriptive cross‐sectional study was submitted to and approved by the Research Ethics Committee of the School of Dentistry of Ribeirão Preto, University of São Paulo (FORP/USP) (CAAE No. 02198018.5.0000.5419) and the Research Ethics Committee on Human Beings of the Ribeirão Preto Medical School (HCRP/FMRP) (CAAE No. 59992522.1.3001.5440). All volunteers signed an informed consent form agreeing to participate in the study.

### 2.2 Sample

The convenience sample consisted of transgender participants (men and women) who were about to start GAHT in 2023 according to the protocol of the Gender Diversity Gynecology Clinic (GDIG) at the Human Reproduction Center of the Department of Gynecology and Obstetrics, Faculty of Medicine of Ribeirão Preto, FMRP/USP. Participants were invited to join the study after completing their initial consultation with a Gynecology resident physician, as per the protocol of the clinic. Following the participants' consultation, the researcher dentist made a formal verbal invitation and demonstrative explanation in a printed guide. All dental assessments were conducted in the dental chair available at the outpatient clinic. The researcher dentist, who was exclusively responsible for these evaluations, was trained and calibrated in assessing TMD based on symptom history and clinical examination as well as in performing clinical evaluations for periodontitis.

### 2.3 Inclusion and Exclusion Criteria

This study included transgender individuals over 18 years old and patients at the AING. Exclusion criteria included participants with cognitive limitations that hindered comprehension of the research instruments, use of analgesics and/or non‐steroidal anti‐inflammatory drugs (NSAIDs) and/or opioids within 24 h before the assessments, participants with reports of head and neck trauma, a history of plastic surgeries or orofacial harmonisation procedures, and those undergoing orthodontic treatment or extensive oral rehabilitation.

### 2.4 Clinical Assessments

Sociodemographic and Economic Questionnaire: A semi‐structured questionnaire collected data on social identification, age, socioeconomic status, alcohol, tobacco and/or drug use, continuous medication use, systemic and oral health conditions, physical activity and time without dental care.

Periodontal Screening and Recording (PSR): Using the Periodontal Screening Recording (PSR) protocol, clinical assessments were performed to evaluate probing depth, bleeding on probing, plaque retention, furcation involvement, mobility, gingival loss and recession. The oral cavity was divided into six sextants, covering teeth 17–14, 13–23, 24–27, 37–34, 33–43 and 44–47. The exam was conducted with a World Health Organization (WHO) type 621 probe (WHO‐621 Trinity; São Paulo, SP, Brazil), and the highest score of the sextant was recorded. PSR scores are classified as follows: Score 0 indicates no bleeding on probing, calculus or overhanging margins. Score 1 indicates bleeding on probing but no calculus or overhanging margins. Score 2 refers to bleeding on probing with the presence of calculus and overhanging margins. Score 3 represents the presence of periodontal pockets with a depth of 3.5–5.5 mm. Score 4 indicates the presence of periodontal pockets greater than 5.5 mm. Finally, the symbol * represents the presence of additional problems, such as furcation involvement, mobility, gingival loss and recession.

Diagnostic Criteria for Temporomandibular Disorders (DC/TMD) (Axis 1): This clinical evaluation tool classifies TMD into up to 12 subcategories: myalgia, local myalgia, myofascial pain, referred myofascial pain, arthralgia, headache attributed to TMD, disc displacement with reduction, disc displacement with reduction with limited mouth opening, disc displacement without reduction with limited opening, disc displacement without reduction and without limited opening, degenerative joint disease and subluxation. The DC/TMD has high sensitivity and specificity (≥ 0.86 and ≥ 0.98, respectively) for any TMD‐related pain.

Generalised Anxiety Disorder‐7 (GAD‐7) (Axis 2): The GAD‐7, translated and validated for use in Brazil in 2006 (Pfizer Inc., New York, NY, USA), has 7 items scored from 0 (not at all) to 3 (nearly every day), with a total score ranging from 0 to 21. The frequency of symptoms in the past 2 weeks is considered, allowing for the classification of anxiety levels into minimal (0–4 points), mild (5–9 points), moderate (10–14 points) and severe (15–21 points).

### 2.5 Statistical Analysis

The statistical analysis began with an exploratory analysis of the data related to the studied variables. The variables considered in this study were age (years), weight (kg), height (cm), income, years of education, living arrangements, habits (alcohol consumption, drug use and smoking), physical activity, previous orthodontic treatment, time since last dental visit (months), anxiety (GAD‐7), gingival/periodontal condition (PSR) and TMD (DC/TMD). To evaluate the association between each variable and gender, the chi‐squared test was performed (*p* < 0.05). The t‐test for independent samples was used for variables associated with age, weight, and height. A multivariate logistic regression was then conducted to assess factors associated with the presence of painful TMD and periodontal disease in the trans population. The variables included in the model were gender, alcohol consumption, drug use, smoking, income, physical activity, anxiety levels and time since last dental visit. The odds ratio was calculated from the logistic regression coefficients by exponentiating these coefficients. The program used for the analysis was JASP 0.18.3.

### 2.6 Intervention and Follow‐Up

In addition to data collection for analysis, participants received oral health guidance and were invited for dental follow‐up at FORP/USP for care and clinical treatment by the researcher to improve their oral health. Improvement involved basic supportive therapies to eliminate infection, control periodontal changes through prophylaxis and supra‐gingival scaling, perform restorative procedures and conduct follow‐up and diagnosis of temporomandibular disorders.

## 3 Results

Of the 54 individuals invited, 45 comprised the study sample. Among these, 23 (51.11%) identified as trans men, one (2.22%) non‐binary individual and 21 (46.66%) identified as trans women. The data from the non‐binary participant were analysed together with those of the trans men, as this participant required GAHT‐T to develop masculine characteristics. The distribution of sociodemographic and economic characteristics, including age, weight, height, income, years of education, living arrangements, smoking habits and alcohol consumption, as well as physical activity and orthodontic treatments, are presented in Table 1. The chi‐squared test found no significant statistical difference between genders. Additionally, six (13.33%) participants reported using psychoactive medications, with an equivalent distribution between genders. Four trans women (19.04%) reported being HIV‐positive and using antiretroviral therapy. The assessment using the GAD‐7 anxiety scale revealed that 19 (42.22%) participants exhibited mild anxiety symptoms (Table 1).

**TABLE 1:** Clinical and sociodemographic characteristics of a transgender and non‐binary population in the pre‐GAHT evaluation.

Characteristics	Trans men (*n* = 24)	Trans women (*n* = 21)	*p*
Age (yeas)	26.38 ± 9.15	24.24 ± 8.49	0.42^a^
Body weight (Kg)	68.99 ± 20.59	73.24 ± 15.79	0.43^a^
Height (cm)	161.83 ± 4.15	173.24 ± 8.49	0.88^a^
Own income	15 62.50	12 57.14	0.71^a^
Years of education			
Basic education (9 years)	3 12.50	5 23.81	0.58^a^
Secondary education (12 years)	19 79.17	14 66.67	
Higher education (16 years)	2 8.33	2 9.52	
Living arrangement			
With family	21 87.50	17 80.95	0.54^a^
Alone	3 12.50	4 19.05	
Habits			
Smoking	12 50.00	7 33.33	0.25^a^
Social drinker	11 45.83	10 47.62	0.90^a^
Physical activity	11 45.83	12 57.14	0.44^a^
Previous orthodontic treatment	12 50.00	6 28.57	0.14^a^
Time since last dental visit (months)	34.33 ± 44.50	33.81 ± 31.27	
GAD‐7			
Normal	10 41.67	5 23.81	0.20^a^
Mild	9 37.50	10 47.62	
Moderate	3 12.50	6 28.57	
Severe	2 8.33	0 0.00	

^a^ *T*‐test for independent samples was used to analyse variables related to age, weight and height. Chi‐squared test was performed for other variables. Quantitative data are presented as mean ± standard deviation. Qualitative data are presented as participants per group (frequency in %). A significance level of *p* < 0.05 was considered.

The analysis of the distribution of PSR indices revealed that 34 (75.55%) participants exhibited at least bleeding on probing and the presence of calculus, requiring some form of periodontal intervention; however, there was no statistically significant difference between genders (*p* > 0.05). Fifteen (33.33%) participants presented other periodontal issues. Regarding the presence of soft tissue lesions, none of the 45 participants evaluated had any lesions (Table 2). The chi‐squared test showed no statistically significant difference in the association between PSR index and gender (*p* > 0.05).

**TABLE 2:** Comparison of Periodontal Screening and Recording (PSR) Index in the baseline assessment of transgender and non‐binary population.

Periodontal Screening and Recording (PSR)^a^	Score	Trans men (*n* = 24)	Trans women (*n* = 21)	*p*
No bleeding on probing, absence of calculus and overhanging margins	0	4 16.67	3 14.29	0.75*
Bleeding on probing, absence of calculus and overhanging margins	1	4 16.67	2 9.52	
Bleeding on probing, presence of calculus and overhanging margins	2	12 50.00	10 47.62	
Presence of periodontal pocket 3.5–5.5 mm	3	4 16.67	5 23.81	
Presence of periodontal pocket greater than 5.5 mm	4	0 0.00	1 4.76	
Presence of other problems like furcation involvement, mobility, gingival recession and loss	*	9 37.50	6 28.57	0.52*

^a^ Data presented by number of participants per group (frequency in %).

* Only the highest value found in each patient was recorded, and the asterisk symbol in PSR represents other periodontal problems. Chi‐squared test associated with gender.

**FIGURE 1:**
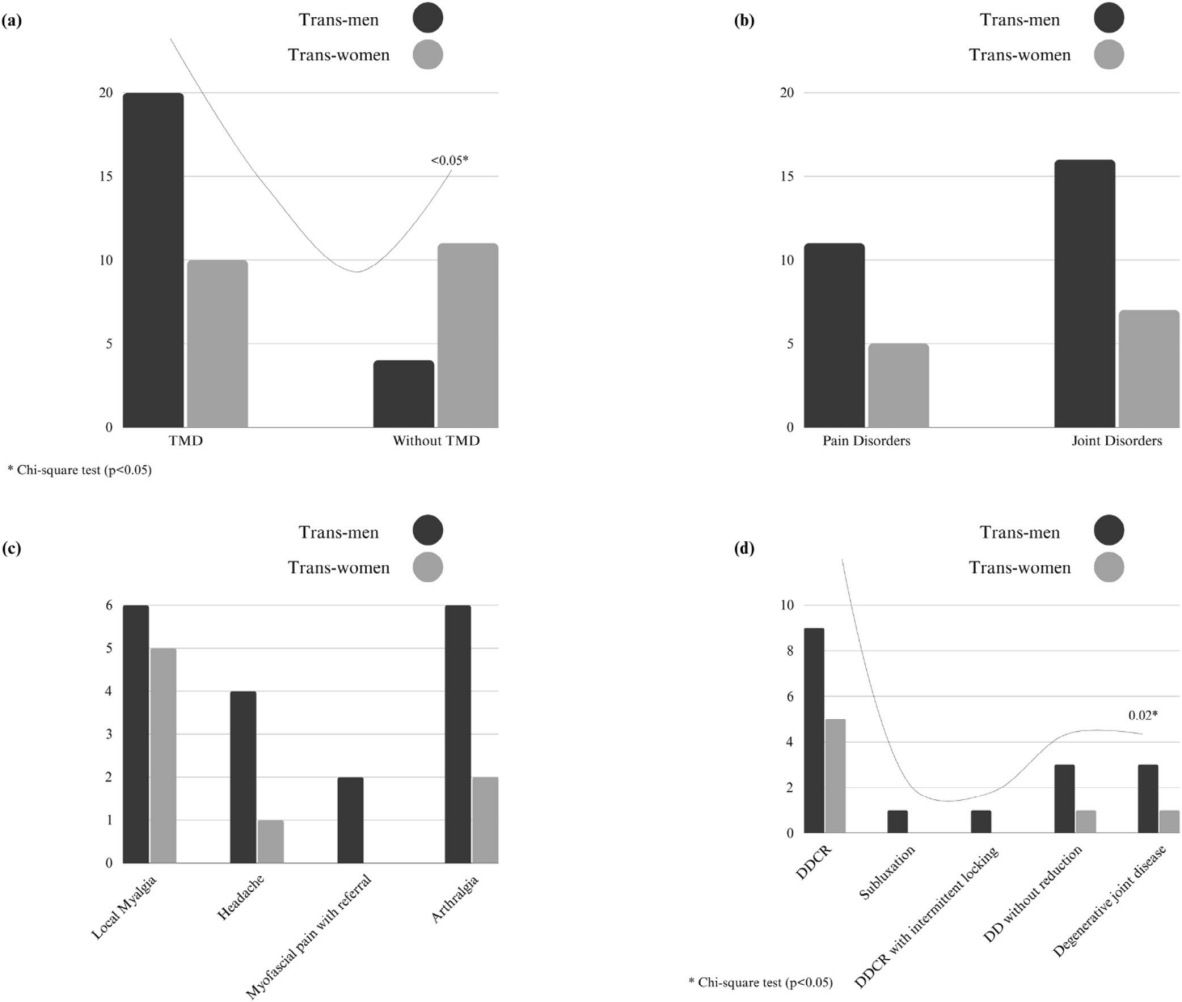
Comparison of TMD diagnoses according to DC/TMD between trans men and trans women: The series of graphs illustrates (a) the presence and absence of TMD; (b) diagnosis of pain disorders versus joint disorders; (c) sub‐diagnosis of pain disorders and (d) sub‐diagnosis of joint disorders. The chi‐squared test revealed statistically significant differences (*p* < 0.05) between genders in both the presence and absence of TMD and in the presence and absence of joint disorders. DD, disc displacement; DDWR, disc displacement without reduction.

Figure 1 presents the TMD diagnostic data according to DC/TMD between trans men and women. The figure shows a series of four graphs illustrating the presence and absence of TMD, the diagnostic groups of pain disorders and joint disorders, specific diagnoses of pain disorders and joint disorders. A chi‐squared test was performed to assess the relationship between gender and these specific diagnoses, revealing statistically significant differences between genders.

Regarding TMD, 30 (66.66%) participants had some form of TMD. The condition was more prevalent among trans men (*p* > 0.05). Detailing the diagnoses, the most frequent were Local Myalgia, present in 11 cases (35.55%), and Disc Displacement with Reduction (DDR) found in 14 cases (46.66%). Additionally, trans men showed a higher prevalence of joint disorders, which was also statistically significant (*p* = 0.02).

Multivariate analysis (Table 3) revealed a significant association between alcohol consumption and gingival/periodontal disease, with a *p*‐value of 0.032 and a positive coefficient, suggesting that alcohol consumption may increase the likelihood of disease occurrence. Moreover, the odds ratio of 1.299 (95% CI: 1.022–1.652) indicates that individuals who consume alcohol are approximately 30% more likely to have gingival/periodontal disease than those who do not. Additionally, anxiety showed a trend towards being a predictor of gingival/periodontal disease, with a *p*‐value of 0.069, but this relationship was not statistically significant at the 5% level. Smoking also showed a trend towards being a significant predictor, with a *p*‐value of 0.073.

**TABLE 3:** Multivariate analysis of periodontal disease predicted by demographic, psychological and lifestyle factors.

Predictors	PSR (Periodontal Screening and Recording)			
	B (SE)	OR	95% CI	*p*
Gender	0.416 (0.248)	1.517	0.932–2.468	0.093
Alcohol consumption	0.262 (0.122)	1.299	1.022–1.652	**0.032**
Drug consumption	0.321 (0.226)	1.379	0.884–2.150	0.155
Smoking	0.203 (0.113)	1.225	0.981–1.531	0.073
Income	0.029 (0.054)	1.029	0.926–1.144	0.586
Physical activity	−0.052 (0.092)	0.949	0.791–1.138	0.574
Anxiety	0.016 (0.009)	1.016	0.998–1.035	0.069
Acess to dental services	0.003 (0.138)	1.003	0.764–1.317	0.978

*Note:* Bold represnts significant at 0.05.

Abbreviations: B, regression coefficient; CI, confidence interval (95%); OR, odds ratio; SE, standard error.

Data from multivariate analysis for the presence of painful TMD (local myalgia, referred myofascial pain, arthralgia and headache attributed to TMD), as presented in Table 4, revealed a significant relationship between gender and the presence of painful TMD, with a *p* value of 0.046 and a positive coefficient. This indicates that certain genders, particularly trans men in the current study, are more likely to report painful TMD. The odds ratio of 1.624 (95% CI: 1.006–2.621) suggests that these trans men are approximately 62% more likely to have painful TMD than trans women. Moreover, alcohol consumption also showed a significant impact on the presence of painful TMD (*p* = 0.049), with an odds ratio of 1.263 (95% CI: 1.000–1.596), indicating that individuals who consume alcohol are approximately 26% more likely to have painful TMD than those who do not. Anxiety was also identified as a significant predictor of painful TMD, with a *p*‐value of 0.043 and an odds ratio of 1.185 (95% CI: 1.000–1.036). This suggests that an increase in anxiety levels is associated with an approximate 18% increase in the likelihood of developing painful TMD.

**TABLE 4:** Multivariate analysis of painful TMD predicted by demographic, psychological and lifestyle factors.

Predictors	Painful TMD (*N* = 16) versus non‐painful TMD (*N* = 29)			
	B (SE)	OR	95% CI	*p*
Gender	0.485 (0.243)	1.624	1.006–2.621	**0.046**
Alcohol consumption	0.234 (0.119)	1.263	1.000–1.596	**0.049**
Drug consumption	0.366 (0.221)	1.443	0.936–2.225	0.097
Smoking	0.192 (0.111)	1.212	0.974–1.508	0.084
Income	0.026 (0.053)	1.027	0.925–1.140	0.614
Physical activity	−0.044 (0.091)	0.956	0.799–1.143	0.626
Anxiety	0.018 (0.009)	1.185	1.000–1.036	**0.043**
Acess to dental services	−0.009 (0.137)	0.990	0.756–1.295	0.945

*Note:* Bold represents significant at 0.05.

Abbreviations: B, regression coefficient; CI, confidence interval (95%); OR, odds ratio; SE, standard error.

Other variables, such as drug consumption, smoking, income, physical activity and access to dental services, did not have a statistically significant association with painful TMD. These results highlight the complexity of TMD aetiology and the need for multidisciplinary approaches that consider both biological and psychosocial factors in the treatment and prevention of this condition.

**FIGURE 2:**
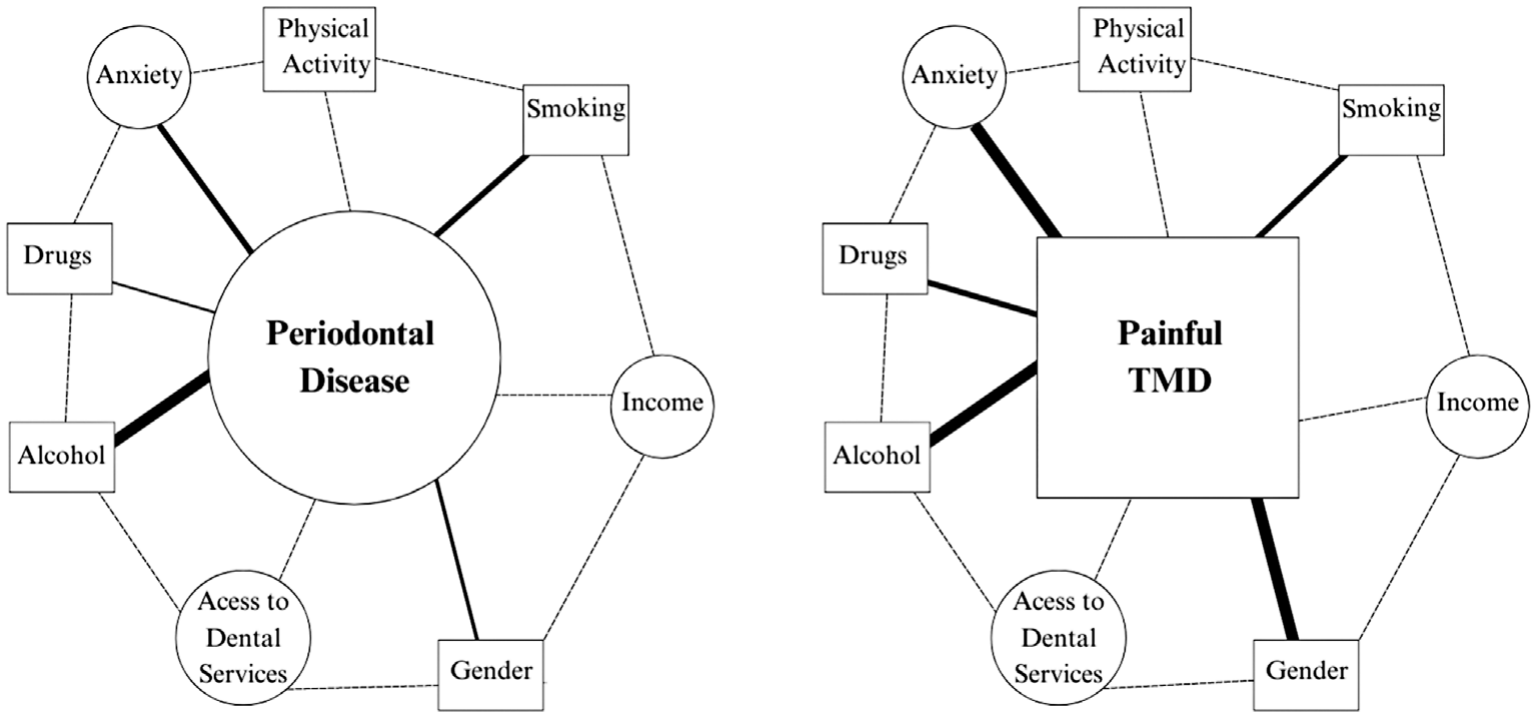
The network model of periodontal disease (left) and that of painful TMD (right). The associations of periodontal disease/painful TMD with gender, alcohol consumption, drug consumption, smoking, income, physical activity, anxiety and access to dental services. The square nodes represent continuous variables; the circular nodes represent categorical variables. Thicker and darker lines indicate stronger associations.

Figure 2 presents a network model that represents the associations between different variables and the analysed oral health conditions. On the left side of the figure, the model for gingival/periodontal disease shows how factors such as gender, alcohol consumption, drug use, smoking, income, physical activity, anxiety and access to dental services are interconnected. On the right side, the corresponding model for painful TMD illustrates how these same factors are associated with the occurrence of pain associated with TMD. In this model, square nodes represent continuous variables, whereas circular nodes indicate categorical variables. The lines connecting the nodes vary in thickness and intensity, reflecting the strength of the associations. Thicker lines indicate stronger relationships between the variables and the condition in question, allowing for visualisation of the complex interactions between various factors and the analysed oral health conditions.

## 4 Discussion

This study reveals a higher prevalence of periodontal health problems and temporomandibular disorders (TMD) signs compared to cisgender counterparts. This highlights a critical need for targeted oral health interventions, explained by necessary periodontal intervention, and systemic changes to improve healthcare access, as participants reported an average of nearly 3 years without visiting a dentist. TMD was also observed, with painful TMD being associated with gender, alcohol consumption and anxiety. The findings align with prior research documenting the disproportionate health challenges faced by transgender individuals due to biopsychosocial factors interconnected through structural stigmatisation rooted in discrimination. Such results support the hypothesis that, even before starting GAHT, social and emotional stressors can exacerbate oral health issues, underscoring the need for integrated approaches in the care of the transgender population.

The oral health of trans people warrants urgent attention, given the alarming prevalence of periodontal issues in this population. Trans and gender non‐conforming individuals are part of a marginalised group that continues to face significant healthcare inequalities [16, 35]. In this study, the Periodontal Screening and Recording (PSR) method was used to evaluate periodontal health by assessing probing depth, bleeding on probing, plaque retention, furcation involvement, mobility, gingival loss and recession. The PSR distribution showed that 66.70% of trans men and 76.19% of trans women had PSR scores between 2 and 4, indicating a need for periodontal intervention. These findings surpass the results of previous studies, such as Manpreet et al. (2022), where 55% of trans participants had a PSR score of 2–4. Furthermore, 16.67% of trans men and 28.57% of trans women in the current study had periodontal pockets deeper than 3.5 mm, a condition comparable to other studies, which found similar rates among transgender participants. The current findings highlight the higher prevalence of periodontal problems in the trans population compared to the general population, as reported in national surveys like the Brasil Sorridente project, the grouping of PSR 2–4 indices suggests a general need for periodontal intervention of 48%, whereas the specific analysis of periodontitis indices (PSR 3 and 4) highlights a higher prevalence of periodontitis in the trans population compared to the findings of the SB Brazil study (19.4%) (Ministério da Saúde, 2011) [22].

After recruiting 54 participants, the final sample included 24 trans men (one of whom was non‐binary but categorised statistically as a trans man) and 21 trans women, reflecting a near‐even gender distribution (53% and 46%, respectively). This differs from prior studies reporting a predominance of trans women, possibly due to the inclusion criterion requiring participants to be hormone‐free for 6 months, a criterion often unmet by trans women who self‐medicate with hormones [35, 39, 40]. Substance use among trans people is notably higher compared to cisgender people, with smoking rates approximately 40% higher [32]. In this study, half of trans men and a third of trans women reported smoking, exceeding national averages (16.2% for men, 9.8% for women) (Brazilian Institute of Geography and Statistics [IBGE], 2019). Similarly, 50% identified as social drinkers, a figure significantly higher than the general population's 26.4%. Smoking, aside from worsening periodontal disease, affects hormone therapy due to thrombosis risk, leading to recommendations to quit at the start of GATH [41], often leading to quitting recommendations. Combined with smoking, it poses a major risk factor for oral cancer [42].

This study higher substance use rates than a previous study by [26] with trans people, which reported that 25% smoked and 20.5% consumed alcohol. High anxiety levels, due to mental health issues and prejudice, are prevalent. In terms of previous orthodontic treatment, half of the trans men reported having undergone such treatment, while less than one‐third of the trans women shared this experience. Access to dental services was notably limited, with participants reporting an average of 3 years since their last dental visit. Research highlights significant barriers trans individuals face in accessing and maintaining healthcare services [22, 23, 43] including oral healthcare. Despite initiatives promoting comprehensive care for gender minorities, challenges persist [22, 44], including limited knowledge among healthcare professionals and prevalent transphobia. These factors contribute to reluctance in seeking care [43, 44, 45]. During follow‐up, participants reported fear of seeking dental care, which may stem from stigma, shame for not adhering to heteronormative standards, internalised homonegativity and the concealment of gender identity [28, 29]. These fears may be exacerbated by negative interactions with healthcare professionals, such as incorrect use of pronouns or failing to respect their social names [34, 36].

The psychological burden on trans individuals, driven by minority stress, further compounds their health challenges. A higher prevalence of mental health disorders, including anxiety and depression, has been consistently documented in the trans population [28, 29]. In this study, approximately 58% of trans men and 76% of trans women exhibited symptoms of anxiety, as measured by the Generalised Anxiety Disorder (GAD‐7) questionnaire. These findings align with prior research, which reported similar or higher rates of anxiety and depression among trans individuals [26, 29]. Multivariate analysis indicated a significant association between alcohol consumption and gingival/periodontal disease, suggesting increased disease risk. Alcohol compromises immune response and exacerbates inflammation, making infections more likely. Anxiety and smoking also emerged as predictors of periodontal disease. The GAD‐7 is a Patient‐Reported Outcome Measure (PROM) [46] with seven questions for assessing Generalised Anxiety Disorder symptoms. It provides valuable insight into patients' anxiety from their perspective. Emotional stress from prejudice can lead to neglect of oral health, and a lack of oral health information is common [24, 28, 36]. For example, some are unaware that bleeding gums indicate poor health or that dental floss should be used even with bleeding [36]. Interventions focused on reducing alcohol and tobacco use and managing anxiety are essential for improving both periodontal and overall health [31, 32, 41]. The prevalence of TMD in this study was also alarming, particularly among trans men, with over 83% affected. This is significantly higher than the prevalence observed in cisgender populations [47, 48, 49].

Previous literature associates oestrogen and other sex hormones with TMD pathogenesis, as hormonal fluctuations may contribute to joint inflammation and pain [9, 10], demonstrating that sex hormones play a role in pain mechanisms and TMD pathogenesis [12, 13]. The role of sex hormones in the development of TMD is well documented, with exogenous oestrogen use in cisgender women being associated with an increased risk of TMD [18]. However, in this study, social and emotional stressors—including minority stress, discrimination and stigmatisation—emerged as likely contributors to this heightened prevalence. Trans men, like trans women and other LGBTQ+ individuals, experience social vulnerability that may increase painful TMD prevalence [49, 50]. Factors such as gender incongruence and transphobia heighten psychological distress, contributing to TMD development [27, 29]. Symptoms of fibromyalgia are six to eight times more common among trans individuals than in the general population [50]. Common diagnoses include local myalgia and disc displacement with reduction (DDR), where the TMJ disc displaces during mouth opening but returns upon closure, often accompanied by a clicking sound [30, 47, 48, 49]. Joint disorders are more prevalent among trans men, potentially due to hormonal effects on joint structure [51]. Multivariate analysis identified alcohol consumption and anxiety as significant predictors of painful TMD, with trans men exhibiting higher odds compared to trans women. These findings suggest that behavioural and psychosocial factors play a critical role in exacerbating oral health conditions in this population.

The present research analysed the oral conditions in trans individuals before starting Gender Affirming Hormone Therapy (GAHT). Periodontal examinations revealed concerning levels of gingivitis and periodontitis, corroborated by other studies, highlighting the need for attention. Analysis results revealed that biopsychosocial variables significantly influence the presence of gingival/periodontal disease and painful temporomandibular disorder (TMD) within the transgender population. Multivariate logistic regression analysis indicated that gender, alcohol consumption, and anxiety were significantly associated with painful TMD, while alcohol consumption was notably associated with gingival/periodontal disease. These findings underscore the critical intersection of these factors affecting the oral health of trans individuals, emphasising social vulnerability as a central element.

The behaviours and attitudes of healthcare professionals can also contribute to these barriers, as a lack of knowledge about transgender health, combined with social prejudices, can result in inadequate care or discrimination. It is essential for healthcare professionals to adopt a biopsychosocial approach to care, recognising the interaction between social determinants, mental health and biological factors in shaping oral health outcomes. Therefore, it is crucial to develop interventions that consider these social and emotional determinants, promoting more inclusive and effective care for the trans population and highlighting critical areas for preventive and supportive health interventions.

## 5 Conclusion

The study concludes that the oral health of trans individuals prior to GAHT is significantly compromised due to both biological factors and social–emotional vulnerabilities, showing a high prevalence of temporomandibular disorders (TMD) and the necessity for periodontal treatment, reinforcing the urgency for inclusive public health policies that address the specific needs of this population and ensure equitable access to care.

## Author Contributions

A.M.M.: sample analysis, data interpretation, study conception and manuscript writing; A.M.M., J.K.T. and M.O.M.: sample analysis, statistical analysis, manuscript writing and revision; L.V.M. and L.A.S.L.: data curation, supervision and manuscript revision; F.C.L.‐O., L.V.M., L.A.S.L. and M.D.S.‐N.: final manuscript revision, supervision and data interpretation; F.C.L.‐O., L.V.M. and L.A.S.L.: statistical analysis support, data interpretation and final manuscript revision; J.F.M.‐C.: studyonception, study design, data interpretation, supervision, data curation and manuscript revision.

## Ethics Statement

This study was conducted at the School of Dentistry of Ribeirão Preto, University of São Paulo (FORP/USP), Brazil, in the year 2023. The research was conducted in accordance with the Declaration of Helsinki and was approved by the Research Ethics Committee of the School of Dentistry of Ribeirão Preto (FORP/USP) under protocol number 02198018.5.0000.5419 and the Research Ethics Committee on Human Beings of the Hospital das Clinicas and the Faculty of Medicine of Ribeirão Preto (HCRP/FMRP) under protocol number 59992522.1.3001.5440.

## Consent

The authors declare that the informed consent obtained from study participants was written. All participants provided written informed consent after receiving a detailed explanation of the study.

## Conflicts of Interest

The authors declare no conflicts of interest.

## Peer Review

The peer review history for this article is available at https://www.webofscience.com/api/gateway/wos/peer‐review/10.1111/joor.13973.

## Data Availability

The data that support the findings of this study are available from the corresponding author upon reasonable request.
